# Advanced Chronic Kidney Disease (CKD) in a Patient With Alstrom Syndrome

**DOI:** 10.7759/cureus.60334

**Published:** 2024-05-15

**Authors:** Moeed Ahmed, Abdul R Ahmed, Rana A Farman

**Affiliations:** 1 Nephrology, Northwestern University, Chicago, USA; 2 Biochemistry, Lahore Medical and Dental College, Lahore, PAK; 3 Psychiatry, The Research Foundation for the State University of New York, Albany, USA

**Keywords:** cataract, genetics, alms1 gene, ckd, alstrom syndrome

## Abstract

Alstrom syndrome is an autosomal recessive disease. It affects multiple systems, including cardiovascular, renal, endocrine, and eyes. Our patient is a 25-year-old female who presented with elevated creatinine. Her past medical history was significant for hypothyroidism, polycystic ovarian syndrome, blindness, cataracts, hearing loss, and heart problems. She had genetic testing done that revealed that she was homozygous for the ALMS1 gene and was diagnosed with Alstrom syndrome. She was followed by nephrology in the clinic and had chronic kidney disease (CKD) stage V. The patient traveled to Italy and was lost to follow-up.

## Introduction

Alstrom syndrome is a rare condition. It is inherited as an autosomal recessive disease. It can affect various systems in the body, including cardiovascular, renal, and endocrinal and eyes. This syndrome was first described by Carl-Henry Alström in 1959 as a progressive retinal degeneration, obesity, neuronal hearing loss, and insulin resistance [1]. About 1,200 cases of Alstrom syndrome have been identified worldwide [2]. At this time, there is no specific therapy for the disease. It affects the males and females in equal numbers. This condition has a poor prognosis, and cardiovascular and end-stage renal diseases (ESRDs) are the major causes of death. 

An abstract of this case was presented at NKF Spring Clinical Meetings 2023 (April 11-15), and no funding has been received yet for this article.

## Case presentation

A 25-year-old Italian female, with a past medical history of hypothyroidism, polycystic ovarian syndrome, cone-rod dystrophy, blindness, cataract, nystagmus, hearing loss, and enlarged heart, presented to the emergency department after being found to have elevated creatinine of 6.9 mg/dL on outpatient blood workup. Baseline creatinine was unknown. A few days earlier, the patient underwent phacoemulsification for a cataract in her left eye. One day after surgery, the patient had a fever, nausea, and flank pain. Her symptoms resolved within four days. She was asymptomatic at the time of presentation.

The vitals were blood pressure of 134/86 mmHg, heart rate of 85 bpm, and respiratory rate of 16. Her height was 149.9 cm, and her BMI was 30.5 kg/m^2^. Her physical exam was negative for any abnormal heart or lung sounds. The abdomen was soft and non-tender, and bowel sounds were present. There was no lower extremity edema.

Family history: Maternal uncles had ESRD. Father was a carrier of the ALMS1 gene. A review of the family pedigree indicated that her parents were consanguineous. Her parents were first cousins. Her mother was a cousin of her father. She had two maternal uncles - both have decreased vision since birth, and one had nystagmus. One of the uncles with the most profound nystagmus had died. She had one maternal aunt with decreased vision and nystagmus since birth.

At the age of two or three, the patient was diagnosed with achromatopsia vs cone-rod dystrophy. She was seen by an ophthalmologist (about four years before admission) who suspected Alstrom syndrome and referred her for genetic testing. The patient had genetic testing done, but she and her mother were not aware of the results. At the age of six months, the patient had cardiac catheterization for an enlarged heart.

Home medications included levothyroxine and metformin for weight loss, which was recently discontinued by the primary care physician (PCP).

Lab results are shown in Table 1.

**Table 1:** Lab results Na=sodium, K=potassium, Cl=chloride, CO2=bicarbonate, BUN=blood urea nitrogen, Cr=creatinine, EGFR= estimated glomerular filtration rate, Ca=calcium, Hb=hemoglobin, PTH=parathyroid hormone

Test	Result	Reference Range
Na (mmol/L)	138	133-146
K (mmol/L)	4.1	3.5-5.1
Cl (mmol/L)	101	98-109
CO2 (mmol/L)	18	21-31
BUN (mg/dL)	98	2-25
Cr (mg/dL)	6.93	0.6-1.3
EGFR (mL/min/1.73 m 2 2)	8	> 60
Ca (mg/dL)	9.9	8.3-10.5
Glucose (mg/dL)	84	65-100
Hb (g/dL	10.5	11.6-15.4
P (mg/dL)	6.8	2.5-5.0
PTH (pg/mL)	380	12-88

Further workup revealed bland urinalysis and urine protein to creatinine ratio of 1.1 mg/mg. LFTs were checked a few days before admission, which were within a normal range, except serum aspartate aminotransferase (AST), which was low. Glycosylated hemoglobin (HbA1c) was 5.2%. Kidney ultrasound showed diffuse echogenic but non-atrophic kidneys. TTE showed a dilated left ventricle, mild left ventricular hypertrophy, and a left ventricle ejection fraction of 53%.

The patient was diagnosed with chronic kidney disease (CKD) stage 5. She did not have any urgent indications of dialysis. A kidney biopsy was not done as it was unlikely to change the management, and the risks of biopsy seemed to be greater than the benefits. She was managed with medical treatment for CKD and was started on sodium bicarbonate, sevelamer, and cinacalcet for metabolic acidosis, hyperphosphatemia, and secondary hyperparathyroidism, respectively. Dialysis option education was offered but refused by the patient's mother. It must be mentioned here that the patient seemed to lack decision-making capacity, and most of the communication regarding her medical condition and care was done with her mother. The patient was discharged home in stable condition after almost two days. Her creatinine was 7.7 mg/dL on discharge day. Discharge medications included sevelamer, cinacalcet, and sodium bicarbonate.

The patient followed up in the nephrology clinic. Genetic testing was reviewed, which revealed that she was homozygous for the ALMS1 gene and was diagnosed with Alstrom syndrome. Test results are as as follows:

Primary Findings

(1) Pathogenic variant: ALMS1 c.9676_9682delinsTA, p.(Arg3226*); homozygous (2) Variant of uncertain significance (VUS): ALMS1 c.11414G>C, p.(Arg3805Thr); homozygous

Secondary Findings

(1) Carrier, pathogenic variant: OCA2 c.593C>T, p.(Pro198Leu); heterozygous; recessive condition (2) VUS: SEMA4A c.808A>G, p.(Lys270Glu); heterozygous; recessive condition

The remaining 348 genes analyzed were negative. She underwent cataract surgery. She got a Holter monitor, which showed non-sustained ventricular tachycardia. She was recommended to see an expert in inherited cardiomyopathy. 

Creatinine soon increased to 9.97 mg/dL. She was referred for dialysis education. She eventually needed a kidney transplant. She traveled to Italy and was lost to follow-up.

## Discussion

Alstrom syndrome is caused by a mutation in the ALMS1 gene. It is an autosomal recessive condition, which means that a person has to inherit two copies of the mutated gene in order to have the disease [3]. Pathophysiology is possibly linked to the ALMS1 protein. ALMS1 protein is found in cilia, and it has been suggested that the absence of protein leads to impairment in the formation of cilia [4].

Alstrom syndrome can affect different systems in the body. Some of the common complications are obesity and early-onset diabetes. Some individuals have growth retardation. Heart failure due to dilated cardiomyopathy is a common complication. It occurs in about 60% of cases and is the most common cause of death [5,6]. A 2012 study suggests that patients with Alstrom syndrome should be evaluated for classical coronary artery disease risk factors and investigated specifically to exclude coronary artery disease [7]. Children affected with Alstrom syndrome can develop vision abnormalities, especially cone-rod dystrophy in the first two years of life. Hearing complications include sensorineural hearing loss and otitis media. Chronic active hepatitis, steatohepatitis, hepatic fibrosis, and cirrhosis are commonly found on liver biopsy [8]. Renal complications are also common and include end-stage renal disease and renal cysts. There is no specific treatment for Alstrom syndrome, and the treatment is focused on the system that is affected. Dark glasses can slow down retinal degeneration. Hearing aids can help in case of hearing loss. Angiotensin-converting enzyme (ACE) inhibitors and loop diuretics can be used for cardiomyopathy. Dialysis and kidney transplants are the options if patients develop end-stage renal disease.

Differential diagnoses of Alstrom syndrome include Bardet-Biedl syndrome (vision loss, obesity, renal failure), Biemond syndrome type 2 (mental retardation, obesity, short stature, coloboma), Wolfram syndrome (early-onset DM and optic atrophy), Cohen syndrome (obesity and retinal dystrophy), familial isolated dilated cardiomyopathy (DCM), and mitochondrial disorders.

Monogenic CKD refers to diseases caused by rare, pathogenic variants in a single gene. Common genetic factors may influence the age of onset, severity, rate of progression, and extrarenal complications of monogenic diseases, which sometimes have variable expression [9,10].

Renasight is a test used to see if there is a genetic cause for kidney disease or if there is an increased risk of the disease due to family history. The test uses a blood or saliva sample to test 385 genes associated with CKD.

The drug PBI-4050 has been tested for its potential use in treating patients with Alstrom syndrome [11].

## Conclusions

Our case highlights the importance of genetic testing in patients who are diagnosed with CKD at a young age. As genetic testing is available for a number of kidney conditions, physicians should utilize this testing and use it in clinical practice. This will not only help in identifying patients at an earlier age but will also help educate patients who have already been affected. Genetic testing for kidney diseases may also help reduce the need for a kidney biopsy, which is an invasive procedure. Genetic conditions should always be considered as the cause when young patients are diagnosed with CKD, and physicians should consider genetic testing for such patients.
